# The Urgent Need to Address Modifiable Risk Factors to Prevent Young-Onset Colorectal Cancer in Mexico

**DOI:** 10.7759/cureus.112604

**Published:** 2026-07-13

**Authors:** Luis A Márquez-Licona, Miguel A Pardiño-Vega, Norma E Herrera-Gonzalez

**Affiliations:** 1 Molecular Oncology Laboratory, Instituto Politécnico Nacional, Mexico City, MEX; 2 Higher School of Medicine, Instituto Politécnico Nacional, Mexico City, MEX

**Keywords:** colorectal cancer, early-onset colorectal cancer, lifestyle risk factors, mexico, obesity

## Abstract

Colorectal cancer (CRC) represents a major cause of morbidity and mortality worldwide and has traditionally been considered a disease of older adults. However, in recent decades, there has been a sustained increase in incidence and mortality among younger populations, particularly in low- and middle-income regions such as Latin America. This article aims to provide an updated overview of CRC epidemiology in Mexico, with emphasis on early-onset disease and modifiable lifestyle-related risk factors. A narrative review of epidemiological studies, population-based reports, and regional case-control analyses was conducted. The reported evidence demonstrates a marked increase in CRC incidence and mortality in Mexico over the last two decades, with a disproportionate impact on adults under 50 years of age. Obesity, sedentary behavior, high intake of red, pork, and processed meats, alcohol consumption, and smoking were consistently associated with increased CRC risk, while diets rich in fiber, fruits, and physical activity showed protective effects. In conclusion, CRC in Mexico reflects an epidemiological transition driven largely by lifestyle changes and social inequities. Addressing modifiable risk factors through prevention strategies and public health policies is essential to curb the rising burden of CRC, particularly among younger and economically active populations.

## Introduction and background

Colorectal cancer (CRC) represents a major public health challenge, currently being the third most frequently diagnosed cancer and the second leading cause of cancer death worldwide. It is defined as a malignant proliferation of cells originating in the epithelial cells of the colon or rectum mucosa, characterized by genetic and epigenetic alterations that result in uncontrolled cell growth and the potential invasion of nearby tissues or the development of distant metastases [1].

Historically, this disease has predominantly affected individuals aged over 50 years. However, in recent decades, a sustained increase in incidence has been documented among younger adults (20-49 years), both in developed countries and in Latin America [2,3]. This phenomenon, which has been identified as early-onset colorectal cancer (EOCRC), defined as that diagnosed in people under 50 years of age, represents one of the most significant epidemiological challenges of today. It has been associated with the adoption of a "Western lifestyle," characterized by obesity, physical inactivity, high consumption of red and processed meats, tobacco use, alcohol consumption, and sugary drinks, with an associated increased risk of between 50% and 100% (OR ~1.5-2.0). This dietary pattern, particularly the intake of processed meats, has also been linked to dysbiosis of the intestinal microbiome and genotoxic damage [4].

In Latin America, the situation is particularly complex; incidence and mortality have increased steadily, registering a 20.56% increase in the mortality rate between 1990 and 2019. Diagnosis is often made at advanced stages, and coverage of preventive programs is limited, which negatively impacts survival and increases the economic burden on health systems [5]. Rapid urbanization and transformation in occupational structures have been associated with a marked decline in habitual physical activity, contributing to increasingly sedentary behavioral patterns.

In Mexico, CRC ranked third in cancer incidence but first in cancer-related mortality in 2022, according to the Global Cancer Observatory (GLOBOCAN) of the International Agency for Research on Cancer (IARC), with more than 16,082 new cases and 8,283 deaths recorded that year, surpassing breast (8,195), lung (7,808), liver (7,673), and prostate cancer (7,358). This mortality burden reflects national aggregate data for both sexes; regional analyses indicate that the highest mortality rates occur in northern Mexico, where the population is predominantly urban. Notably, this trend has been paralleled by a rising proportion of cases among adults under 50 years of age, a phenomenon further examined in the following section [6].

Changes in the traditional Mexican diet, characterized by foods rich in corn, legumes, and fruits, have contributed significantly to this phenomenon. This nutritional transition has been associated with a high consumption of added sugars and saturated fats, which, in turn, has been linked to 75.2% of adults in Mexico being overweight or obese, conditions associated with an increased risk of several chronic diseases [7].

## Review

Search strategy

A narrative literature search was conducted using PubMed, SciELO, and ResearchGate, combining terms including "colorectal cancer," "young-onset colorectal cancer," "risk factors," "obesity," "diabetes," "alcohol," "tobacco," "diet," "colorectal cancer in Mexico," and "colorectal cancer statistics." The search prioritized articles published between 2019 and 2026. However, earlier publications (pre-2019) were included when necessary to establish fundamental concepts, definitions, or mechanistic evidence linking specific risk factors to CRC. Articles published in English or Spanish specifically were considered, with a primary focus on populations from the American continent and a particular emphasis on epidemiological data from Mexico and epidemiological transition patterns in the broader Latin American region. National statistics of Mexico referenced throughout this review correspond to data reported through 2022-2024. Studies conducted outside Mexico were incorporated selectively to provide mechanistic, biological, or comparative context supporting the interpretation of risk factors within the Mexican population, rather than as direct epidemiological substitutes for national data.

Early-onset colorectal cancer (EOCRC): an emerging global crisis

The increase in the incidence and mortality of CRC among young adults is alarming. Future projections underscore the severity of this situation, estimating that the global incidence of CRC will increase considerably by 2030. Specifically, in the 20-34 age group, a 90% increase in colon cancer and a 124.2% increase in rectal cancer are projected; in the 35-49 age group, a 27.7% increase for colon cancer and a 46% increase for rectal cancer are expected [3].

In the United States of America, new cases of CRC in patients younger than 55 years increased from 11% to 20% of the total between 1995 and 2019 [8]. Unlike older age groups, where mortality has decreased, the incidence has increased more sharply in young adults (3% annually in the 20-49 age group) than in middle-aged adults (0.4% annually between 50 and 64 years), with a predominance of tumors in the distal colon and rectum [9].

Epidemiological overview in Mexico

CRC represents a critical public health challenge in Mexico, ranking as the third most common cancer and the leading cause of cancer-related death in the country according to 2022 data. During that year, more than 16,000 new cases and 8,200 deaths were recorded, reflecting a growing trend in which the age-adjusted mortality rate increased from 3.6 to 5.5 per 100,000 inhabitants between 1998 and 2018, a rise that was particularly pronounced among men. Men aged 50 years and older show the highest incidence (50.7 per 100,000) and mortality (27.0 per 100,000) rates nationally [6].

Beyond epidemiological trends, emerging Mexican clinical data reveal that EOCRC also represents a biologically distinct entity. A retrospective cohort study conducted at Hospital General de México (2020-2024), comprising 583 patients with confirmed CRC, found that 35.2% of patients belonged to the EOCRC group, a significantly higher prevalence than the 10-15% reported in population registries in the United States and Europe. These patients were significantly more likely to present with poorly differentiated tumors compared to late-onset colorectal cancer (LOCRC) patients (24% vs. 15%, p=0.020). Notably, 70% of EOCRC cases were diagnosed at advanced stages (III-IV). EOCRC patients received adjuvant chemotherapy more frequently than LOCRC patients (69% vs. 59%, p=0.040), while LOCRC patients underwent adjuvant radiotherapy more often (25% vs. 1.5%, p<0.001). These findings support the characterization of EOCRC as a distinct clinical and pathological entity requiring heightened clinical suspicion and age-adjusted screening approaches in the Mexican population [10].

Determinants of disease risk in Mexico

The risk of developing CRC is multifactorial, involving genetic, environmental, and behavioral components. Family history has been confirmed as an invaluable clinical tool for assessing risk. The presence of a family history of any malignancy is associated with a threefold increase in the risk of developing CRC in the Mexican population (OR = 3.19 (95% CI: 1.81-5.60) [7]. This risk increases progressively with familial burden and is more pronounced in younger individuals, in whom having a parent with CRC can quadruple the risk compared with peers without such a history. In addition to genetic background, region-specific dietary factors emerge as crucial [7,11].

Within the sociocultural and epidemiological context of Mexico, accumulating evidence indicates that the rising incidence of the disease is predominantly driven by dietary patterns, obesity, tobacco use, and excessive alcohol consumption [12]. A population-specific analysis in Mexico has quantified the magnitude of the association between these habits and the risk of developing CRC. For instance, frequent consumption of pork (defined as three or more times per week) has been associated with a more than threefold increase in risk (OR = 3.26 (95% CI: 1.34-7.90)), while frequent beef consumption nearly tripled the risk (OR = 2.95 (95% CI: 1.05-8.26)) [7].

**Figure 1:**
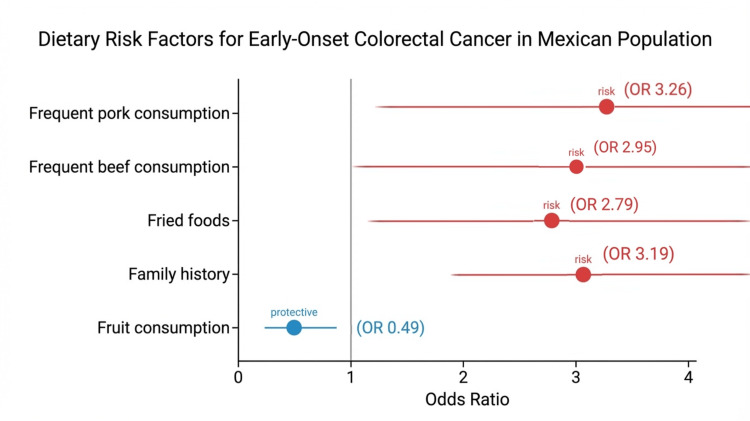
Dietary risk factors and family history of colorectal cancer in the Mexican population. Image created by the authors using Microsoft PowerPoint (Microsoft Corp., Redmond, WA, USA).

In addition to red meats, frequent consumption of regionally typical fried foods was also identified as a significant risk factor, increasing the likelihood nearly threefold (OR = 2.79 (95% CI (1.32-5.89)); an inadequate intake of fruits and vegetables constitutes a significant risk factor, whereas frequent fruit consumption was shown to be protective, reducing the risk of CRC by half (OR = 0.49 (95% CI: 0.28-0.88) (Figure 1) [7]. These findings underscore that, although obesity and reduced fiber intake contribute to the increasing burden of the disease, local dietary patterns, in conjunction with the daily intake of sugar-sweetened beverages, constitute key determinants for understanding and addressing the growing burden of CRC among young individuals in Mexico.

Local dietary patterns

Dietary influences operate through multiple biological and pathophysiological pathways [13]. A dietary pattern characterized by high fat content, low fiber intake, and frequent consumption of red and processed meats constitutes a set of key modifiable risk factors implicated in the development of CRC [14]. Fiber, as a protective agent, disrupts the metabolism of bile acids, thereby reducing their carcinogenic potential in the colon. Fiber-rich foods such as vegetables supply abundant antioxidant compounds (including resveratrol, polyphenols, and phytoestrogens) that help counteract cellular damage; commensal gut bacteria ferment fiber to produce butyrate, a compound with well-documented antiproliferative properties; and finally, fiber actively modulates the composition of the gut microbiota, whose imbalance has been increasingly linked to CRC development [15].

Metabolic and hormonal pathways extend beyond direct effects, as dietary patterns contribute to carcinogenesis indirectly by promoting obesity, which subsequently drives metabolic dysfunction. Conditions such as insulin resistance, persistent low-grade inflammation, and hormonal imbalances involving leptin and adiponectin create an environment conducive to abnormal cell growth and tumor progression [13]. The involvement of bile acids in carcinogenesis is of considerable relevance. Dietary patterns prevalent in Mexico are associated with increased bile acid secretions, particularly deoxycholic acid (DCA), whose elevated concentrations have been demonstrated to exert cytotoxic effects, compromise the integrity of colonic epithelial cells, and induce genomic instability, all of which create favorable conditions for CRC formation [16]. Another highly prevalent sociocultural practice in Mexico is the traditional barbecue gathering, during which a variety of processed and cured meats are routinely prepared by grilling: sausages, bacon, ham, salami, and chorizo. Many of these products may contain curing salts with nitrates, which are commonly used in meat processing for preservation and color stabilization.

The adoption of healthy lifestyle patterns has been consistently demonstrated to attenuate risk [17,18]; adherence to the Mediterranean diet reduces the risk of CRC by 21%. Fish consumption reduces risk by 12%, dietary fiber intake (>20 g/day) reduces risk by 25%, and high dairy consumption reduces risk by 26% in men. Omega-3 polyunsaturated fatty acids (present in fish) have anti-inflammatory effects and may prevent the progression of obesity and cancer [19].

In Mexico, if current consumption patterns are not modified - where most individuals consume sugar-sweetened beverages and few eat vegetables - the CRC mortality trend will continue to rise, increasingly affecting people of working age. However, the outlook in Mexico remains unfavorable, as nearly 80% of the population consumes sugar-sweetened beverages with each meal on a daily basis [20]. Effective prevention strategies must extend beyond taxation and warning labels on sugar-sweetened beverages.

Obesity

**Figure 2:**
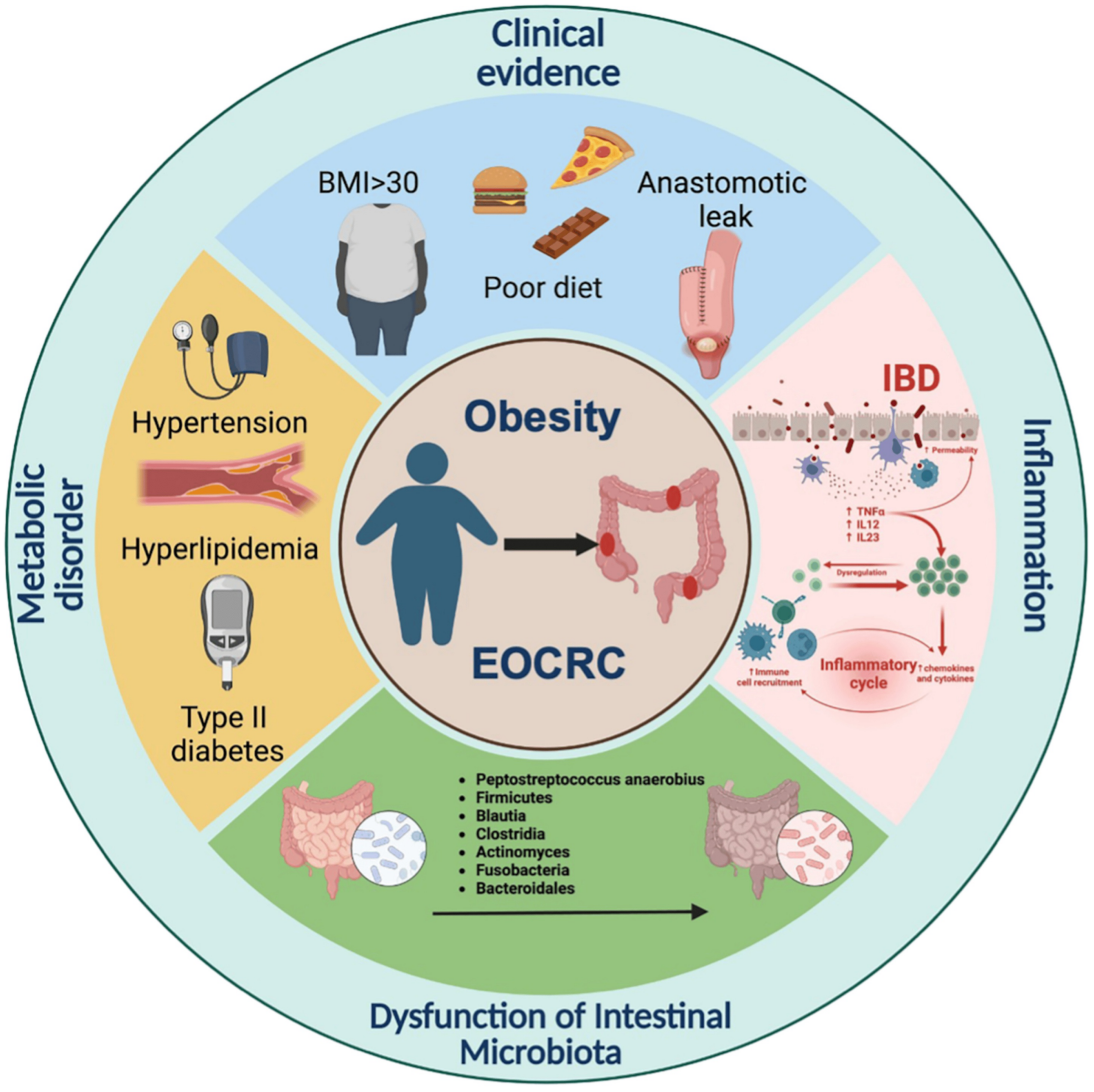
The bridge between lifestyle risk factors and colorectal cancer. IBD: inflammatory bowel disease; EOCRC: early-onset colorectal cancer Image reproduced from [22] under the Creative Commons Attribution License (CC BY 4.0).

Obesity and overweight have been identified as major contributors to the increase in mortality from colon cancer. A body mass index (BMI) greater than 30 kg/m^2^ defines obesity, which significantly increases the incidence of CRC (41% greater probability of CRC onset relative to those maintaining a healthy weight), particularly in men, who appear to be more susceptible than women (likely due to the protective effects of estrogens) (Figure 2) [21,22].

**Figure 3:**
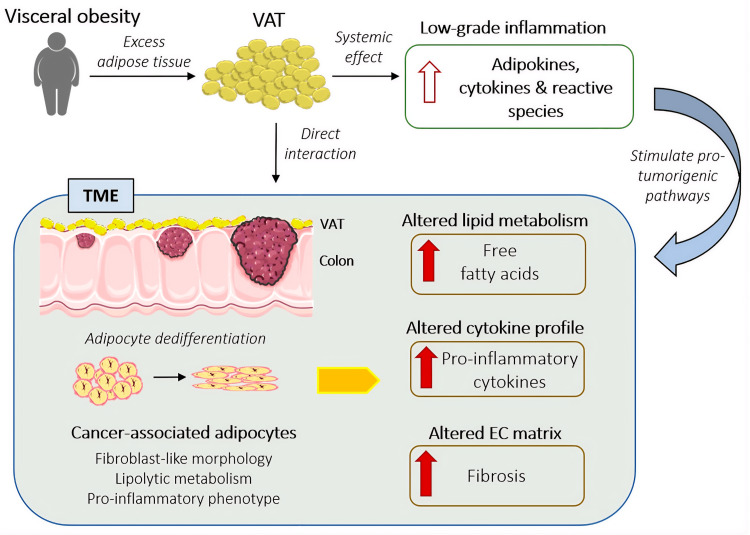
Pathophysiology of colorectal cancer: obesity and sedentary behavior as key factors. VAT: visceral adipose tissue; EC: extracellular Image reproduced from [25] under the Creative Commons Attribution License (CC BY 4.0).

Obesity exerts its carcinogenic effects through chronic low-grade inflammation, insulin resistance, and alterations in the gut microbiota. These processes lead to the release of factors such as insulin-like growth factor 1 (IGF-1), proinflammatory cytokines (TNFα, IL-6, IL-1), and adipokines (leptin), which promote cellular proliferation, angiogenesis, and inhibition of apoptosis, thereby facilitating the progression from normal epithelium to adenomas and ultimately to carcinoma (Figure 3) [23-26].

Beyond body composition, sedentary behavior constitutes an independent and modifiable risk factor for CRC. Regular physical activity has been shown to reduce relative CRC risk by 21% to 27%, with benefits observed across a spectrum of intensities, from vigorous activities such as climbing to moderate ones such as brisk walking. Notably, engaging in one or more hours of exercise per week is associated with a lower prevalence of colonic polyps, and physical activity has also been shown to improve post-diagnosis survival, particularly in stages II and III [19].

The mechanistic link between inactivity and CRC operates partly through visceral adipose tissue (VAT), which (distinct from subcutaneous fat) functions as an active endocrine organ, amplifying pro-tumorigenic signaling even in individuals who do not meet clinical criteria for obesity [27].

Evidence from a systematic review analyzing 12 randomized controlled clinical trials found that physical activity was associated with improved insulin sensitivity in seven of the included studies, suggesting that regular exercise may help counteract the metabolic dysregulation that contributes to cancer progression [28]. Based on this information, sustained physical exercise is associated with a metabolic and hormonal profile less permissive to tumor development, including improved insulin sensitivity and reduced systemic inflammation [28,29].

Diabetes

The convergence of obesity, dietary patterns, and physical inactivity not only elevates CRC risk directly, but it also creates the metabolic conditions for the onset of type 2 diabetes, a disease that compounds that risk through its own independent pathways and is defined as a metabolic disorder characterized by chronic hyperglycemia, caused by insufficient insulin secretion, deficient insulin action, or both. Insulin, as an anabolic hormone, plays a fundamental role in the metabolism of carbohydrates, lipids, and proteins [30].

Diabetes has been identified as an independent risk factor for the development of CRC, supported by evidence showing that patients diagnosed with colon and rectal cancer report a history of diabetes more frequently than the general healthy population [31]. This association is underpinned by a series of pathophysiological mechanisms in which metabolic disturbances inherent to the disease (including hyperglycemia, hyperinsulinemia, and metabolic syndrome) stimulate malignant cell proliferation through the activation of IGF-1 receptors, a pathway that suppresses apoptosis and promotes tumor progression. Likewise, type 2 diabetes generates additional disruptions in the cellular microenvironment, including the overproduction of pro-inflammatory cytokines such as IL-6 and TNF-α, as well as imbalances in leptin and adiponectin levels, which diminish endogenous programmed cell death mechanisms and enhance the invasive capacity of the tumor [31,32].

Hyperinsulinemia activates insulin and IGF receptors, triggering signaling cascades (PI3K/Akt/mTOR and MAPK) that promote the growth, survival, and spread of cancer cells. Meanwhile, hyperglycemia (persistent excess of glucose in the blood) enhances the Wnt/β-catenin pathway (a cellular signaling route that, when abnormally activated, promotes uncontrolled cell division and tumor formation), increasing the expression of vascular endothelial growth factor (VEGF) and stimulating the formation of new blood vessels that nourish the tumor. Finally, diabetic dyslipidemia supplies free fatty acids (non-esterified fatty acids, or NEFA) that activate oncogenic pathways and provide energy for cell proliferation [33].

Alcohol consumption

Alcohol consumption exhibits a dose-dependent relationship with CRC risk: the greater the intake, the higher the associated mortality [34]. Its carcinogenic effect operates primarily through two converging mechanisms: the antifolate effect, whereby alcohol depletes folic acid and thereby compromises DNA synthesis and repair, and the direct mutagenic action of acetaldehyde, a toxic byproduct of alcohol metabolism that acts as a promoter of colonic and rectal carcinogenesis [35]. The male population is disproportionately affected, with a relative risk (RR) of 1.16 even at light consumption levels (under 12.4 g/day), rising to an RR of 1.48 among heavy drinkers in the general population [36].

In the Mexican context, a 2018 study conducted in the northern region of the country reported that among individuals with a family history of CRC, alcohol consumption was markedly high and showed clear sex-based differences: 77% of men reported harmful consumption levels, compared with 35.2% of women classified as at-risk drinkers [36]. This disparity has been linked to socially constructed gender roles, in which alcohol use is more culturally accepted among men and associated with masculinity and risk-taking behavior [36].

Particularly relevant for early-onset CRC, evidence suggests that reducing or eliminating heavy alcohol consumption could lower risk to a degree comparable to having a substantially more favorable genetic profile, underscoring that lifestyle modification can functionally offset hereditary predisposition [37]. Yet this risk remains poorly recognized: only 28.7% of surveyed individuals identified consuming more than one alcoholic drink per day as a CRC risk factor [36], highlighting the urgent need for targeted public health communication.

Smoking

Smoking represents a well-established and independently quantified risk factor for CRC, with carcinogenic effects that operate from the earliest stages of neoplastic development and whose magnitude varies notably by sex, anatomical subsite, and duration of exposure. In a large population-based case-control study (n = 9,206), current smokers showed a 48% higher risk of CRC compared with never smokers (adjusted OR 1.48, 95% CI 1.27-1.72), with dose-response relationships confirmed across pack-year categories [38].

Tobacco smoke contains carcinogens that reach the colorectal mucosa either through the digestive tract or via the bloodstream. These components can damage the expression of key genes and initiate carcinogenesis. A study conducted in a middle- and low-income population (Pasto, Colombia), in which heavy cigarette consumption showed the strongest association with the development of CRC, indicates that smoking is associated with the appearance of small adenomas (benign tumors) during the first 20 years of consumption, acting as carcinogens at very early stages of the disease. A prolonged duration of exposure (20-30 years) is required for the formation of neoplasia [29].

The impact of smoking on CRC risk differs meaningfully by sex and anatomical subsite. In a large-scale six of nine epidemiological analysis of 188,052 subjects, male smokers showed a 39% higher incidence of left-sided colon cancer compared with non-smokers, while female smokers exhibited a 20% higher risk of right-sided colon cancer, with rectal cancer risk in women increasing proportionally with cumulative pack-years [39]. Genetic background further modulates this risk: in individuals with hereditary predisposition (such as patients with Lynch syndrome), tobacco exposure substantially amplifies the likelihood of adenoma development, making smoking cessation especially critical in these populations [34].

Data from hospital-based studies in Mexico reflect these patterns at the local level: while 100% of women surveyed reported not smoking, tobacco dependence was documented in nearly a quarter of men (24.5%), consistent with the same gender-role dynamics that drive differential alcohol consumption in the Mexican population [36].

The quantitative relationship between smoking and genetic predisposition has been formalized through the concept of genetic risk equivalent (GRE): the effect of current smoking on CRC risk is equivalent to carrying a polygenic risk score (PRS) that is 30 percentiles higher than average [38]. This means that abstaining from smoking can functionally compensate for a substantial proportion of genetically predetermined CRC risk [38].

Complementing these findings and considering the broader context of smoking behavior, analyses of CRC development by sex indicate that women who smoke have a 58% higher risk of rectal cancer compared with non-smoking women, whereas in men this increase is 40%. That is, smoking represents a relatively greater risk factor in women than in men [39].

Limitations, recommendations, and future directions

This narrative review has several limitations that should be acknowledged. First, much of the available evidence regarding CRC risk factors derives from observational and retrospective studies, which may be subject to selection bias, recall bias, and residual confounding. In addition, epidemiological data specific to Mexico and Latin America remain limited compared with those available from high-income countries, restricting the ability to establish precise national trends and causal relationships. Another limitation is the heterogeneity among included studies regarding population characteristics, dietary assessment methods, and definitions of lifestyle exposures, which may affect the comparability of findings. Furthermore, because this review is narrative rather than systematic, it does not provide a quantitative synthesis or meta-analytic estimation of risk.

Despite these limitations, the evidence consistently supports the major role of modifiable lifestyle factors in the rising burden of EOCRC in Mexico. Therefore, strengthening prevention strategies should become a national public health priority. Public policies aimed at reducing obesity, limiting the consumption of sugar-sweetened beverages and processed meats, promoting healthier dietary habits, increasing physical activity, and reinforcing tobacco and alcohol control measures are urgently needed. Likewise, improving awareness among both the general population and healthcare professionals regarding early CRC symptoms in younger adults could facilitate earlier diagnosis and improve outcomes.

Future research should focus on large-scale prospective studies specifically designed for the Mexican and Latin American populations to better characterize regional risk factors, sociocultural determinants, and genetic-environmental interactions associated with CRC. Additional investigation into the impact of ultra-processed foods, microbiota alterations, metabolic dysfunction, and environmental exposures may help clarify mechanisms underlying the increasing incidence of early-onset disease. Expanding population-based screening programs and evaluating the cost-effectiveness of initiating screening at younger ages in high-risk populations should also constitute key research priorities. Finally, multidisciplinary strategies integrating epidemiology, molecular biology, nutrition, and public health policy will be essential to effectively address the growing challenge of CRC in younger populations.

## Conclusions

CRC in Mexico has undergone a marked epidemiological transition, shifting from a disease predominantly affecting older adults to an increasingly prevalent condition among younger and economically active populations. This trend has been associated primarily with modifiable lifestyle-related factors, including obesity, sedentary behavior, dietary patterns characterized by low fiber intake and high consumption of sugar-sweetened beverages and processed meats, alcohol consumption, and tobacco use. The convergence of these factors may substantially amplify CRC risk beyond that associated with any single exposure, underscoring the importance of comprehensive prevention strategies.

Addressing these trends requires a coordinated public health response centered on early intervention, health education, and structural policies that facilitate healthier lifestyles, including sugar-sweetened beverage taxation, front-of-package warning labels, and improved access to healthy foods. Furthermore, CRC screening coverage remains limited in Mexico, with no nationwide screening program currently implemented. Strengthening both primary prevention through lifestyle modification and secondary prevention through evidence-based screening strategies for individuals at increased risk will be essential to reducing the future burden of CRC in the Mexican population.
